# Radiotherapy following breast-conserving surgery for screen-detected ductal carcinoma *in situ*: indications and utilisation in the UK. Interim findings from the Sloane Project

**DOI:** 10.1038/sj.bjc.6603945

**Published:** 2007-09-11

**Authors:** D Dodwell, K Clements, G Lawrence, O Kearins, C S Thomson, J Dewar, H Bishop

**Affiliations:** 1 Department of Clinical Oncology, Cookridge Hospital, Hospital Lane, Leeds, LS16 6QB, UK; 2 West Midlands Cancer Intelligence Unit, Public Health Building, The University of Birmingham, Birmingham, B15 2TT, UK; 3 Ninewells Hospital, Dundee, DD1 9SY, UK; 4 Bolton Breast Unit, Royal Bolton Hospital, Minerva Road, Farnworth, Bolton, BL4 0JR, UK

**Keywords:** breast cancer, radiotherapy (RT), ductal carcinoma *in situ* (DCIS), breast-conserving surgery (BCS), the Sloane Project, screen-detected

## Abstract

Use of radiotherapy (RT) after breast-conserving surgery (BCS) for ductal carcinoma *in situ* (DCIS) varies according to country, precedent and prejudice. Results from a preliminary analysis of the data available within the UK Sloane Project can be appreciated in the context of the uncertainty concerning the selection of adjuvant RT following BCS for DCIS. There was a marked geographical variation in the use of RT within the United Kingdom. However, overall, patients with DCIS treated with BCS were significantly more likely to have RT planned (and given) if they had large (⩾15 mm), intermediate or high-grade tumours or if central comedo-type necrosis was present. Unexpectedly, margin width did not appear to have a significant effect on the decision-making process. However, the Van Nuys Prognostic Index did significantly affect the chances of getting planned RT in the univariate analysis, suggesting that clinicians may be starting to use this scoring system in routine practice to assist in decision making.

The Sloane Project was set up to assess the incidence, patterns of care and outcome of screen-detected ductal carcinoma *in situ* (DCIS) within the UK by prospective collection of radiological, pathological, surgical, systemic therapy and radiotherapy (RT) data using specifically designed proforma. All 94 UK NHS breast-screening units are encouraged to participate. Data collection began in April 2003 and continues to date. We report the findings from the start of data collection until June 2006.

There is an increasing use of breast-conserving surgery (BCS) in preference to mastectomy as definitive surgical therapy for DCIS. Over the last decade, a number of randomised controlled trials have confirmed that post-operative whole-breast irradiation (RT) following BCS for DCIS reduces the risk of both *in situ* and invasive recurrence. However, examination of data from clinical trials to date does not readily allow the identification of patients who benefit most from, or conversely do not require, RT. A number of factors, which predict the risk of local recurrence, have been used either alone or in combination to select patients to receive RT. We have investigated the use of RT following BCS for screen-detected DCIS within the Sloane Project database.

## MATERIALS AND METHODS

**Figure 1:**
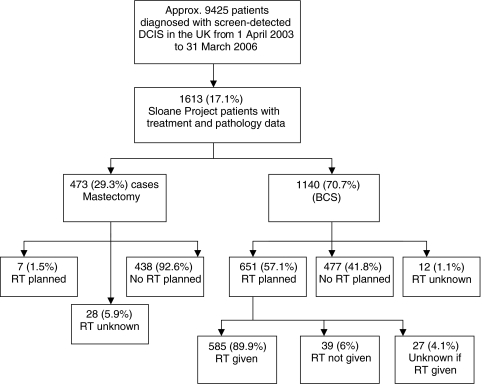
Details of the final surgical treatment received by and adjuvant radiotherapy treatment planned and confirmed as given to patients included in the Sloane Project.

The study group consisted of 1140 patients who underwent BCS for DCIS (either alone or in combination with lobular *in situ* neoplasia and/or atypical ductal hyperplasia) between April 2003 and June 2006 and for whom both treatment and pathology data were available (Figure 1). Data were available from 69 of the breast screening units within the United Kingdom. Margin data were collected on the Sloane Project pathology form. Pathologists were requested to provide information regarding the width of tumour-free tissue as far as possible for each individual radial (i.e. not anterior or deep) margin. For the purposes of the analyses, the smallest margin width following the final therapeutic excision was used for any of the radial margins. If the specimen was not orientated by the surgeon at the time of operation, a single, non-specified margin measurement was provided. If the only margin width given was distance to the deep, superficial or nipple margins, margin status was regarded as ‘unknown’. Radiotherapy treatment was defined as ‘planned’ if, according to the Sloane Project surgical treatment form, the intention was to refer a patient for RT, either with or without endocrine therapy. Radiotherapy treatment was defined as ‘given’ if a Sloane Project RT treatment form recording the details of the RT was submitted or other evidence was provided that the RT treatment had been given.

The planning of RT and actual RT recorded as given were examined in relation to the pathological characteristics of the DCIS and also with pathological assessment of margin status/width. The Van Nuys Prognostic Index (VNPI) (Silverstein *et al*, 1996) was calculated. The VNPI combines three significant predictors of local recurrence: tumour size, margin width and pathologic classification. Scores of 1 (best) to 3 (worst) are assigned for each of the three predictors and then totalled to give an overall VNPI score ranging from three to nine.

Several univariate logistic regression models were fitted to determine which factors predicted whether RT was planned for, or actually received by individual patients. The factors were further examined using a single logistic multivariate regression model (Armitage and Berry, 1994) to examine the effect of forcing each of the factors into the model, after adjustment for the other factors. Both unadjusted and adjusted odds ratios are presented for each level of the different factors compared with the reference baseline category.

## RESULTS

Of the 1613 patients registered within the Sloane Project database, 473 (29.3%) were treated by mastectomy and 1140 (70.7%) by BCS (Figure 1). Overall, in slightly more than half (651 (57.1%)) of the 1140 patients treated by BCS, the intention to give post-operative adjuvant RT was recorded on the surgical treatment form. Five hundred and eighty-five (89.9%) of these 651 cases had a completed RT form returned. Margin width was available for 87.7% of cases, tumour size for 95.4%, the presence or absence of comedo necrosis for 97.5% and nuclear grade for 99.0%. A VNPI could be calculated for 83.2% of patients. Table 1 shows how the intended (planned) and actual (planned and recorded as given) use of adjuvant RT varied with excision margin width, pathological assessment/estimation of the size of the DCIS, the presence of comedo necrosis, DCIS nuclear grade and VNPI.

Table 1 shows that planned use of adjuvant RT was significantly higher for larger tumours (82.9% of patients with DCIS >40 mm in diameter compared with only 45.3% of patients with <15 mm diameter tumours), for tumours with a high nuclear grade (73.1% compared with 17.7% for low-grade DCIS) and for DCIS with central comedo-type necrosis present (69.9% compared with 33.4%). Logistic regression modelling showed that all of these factors were highly significant in both the univariate and multivariate models predicting the planned use of adjuvant RT. This indicates that all three factors are strong independent determinants in the decision-making process, after taking the other factors into account (Table 2).

Planned use of adjuvant RT increased with decreasing excision margin width for tumours where the excision margins were greater than 1 mm; ranging from 55.2% for tumours with margins ⩾10 mm and 64.6% for tumours with a margin width of 1–4.99 mm (Table 1). However, logistic regression analysis showed that margin width did not significantly affect whether or not adjuvant RT was planned (*P*=0.09; Table 2). For 49 tumours with excision margins <1 mm (41.9% of those with margins <1 mm and 4.3% of all cases) no adjuvant RT was recorded as being planned (Table 1). For a further five cases, adjuvant RT was planned but not given, and for six other cases adjuvant RT was planned but it was not known whether or not the treatment had actually been given.

The planned use of adjuvant RT also showed a strong relationship with VNPI, increasing from 27.1% for tumours with a score of 3 or 4, to 68.2% for tumours with a score of 5, 6 or 7 and 79.5% for tumours with a score of 8 or 9 (Table 1). This relationship was observed in a univariate logistic regression model, with patients with a VNPI of 5–7 being 6 times more likely, and patients with a VNPI of 8 or 9 being 10 times more likely to have RT planned compared with patients of VNPI 3 or 4 (*P*<0.001). However, this effect was no longer statistically significant in the multivariate model after adjustment for the factors tumour size, nuclear grade and presence or absence of comedo necrosis (*P*=0.75).

The logistic regression analyses were repeated for the actual use of adjuvant RT. The findings were very similar to those for the planned use of adjuvant RT, with tumour size, comedo necrosis and nuclear grade being the only significant predictors in the multivariate model (all *P*<0.001; data not shown).

**Figure 2:**
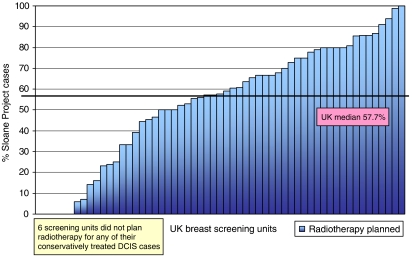
Variation in the planned use of adjuvant radiotherapy with breast-screening unit.

There was wide variation in the use of adjuvant RT across UK breast-screening units, with six units not using adjuvant RT at all, and two units giving adjuvant RT to all of their patients. The median planned use of adjuvant RT was 57.7% (Figure 2).

## DISCUSSION

The number of patients diagnosed with screen-detected DCIS within the United Kingdom annually is around 2900, and approximately 70% of these are treated with BCS (NHS Breast Screening Programme (NHSBSP) and Association of Breast Surgery (ABS) at BASO, 2007). The study group therefore comprised around 12% of all incident cases of DCIS diagnosed during the period April 2003–June 2006 and 17% of those receiving BCS.

DCIS patients treated with BCS were significantly more likely to have adjuvant RT planned, and to have been given adjuvant RT, if their tumours were large (⩾15 mm), if they had either intermediate- or high-grade tumours, or if central comedo-type necrosis was present. This indicates that knowledge about each of these factors informs the decision-making process separately. While there did appear to be a general increase in the planned use of adjuvant RT with decreasing excision margin width for tumours where the excision margins were greater than 1 mm, this factor was not significant in the multivariate modelling and so did not independently predict whether adjuvant RT was planned. The lack of an overall effect for margin width in the multivariate model is possibly due to the high percentage of cases who were planned not to receive RT with margins <1 mm, where this may have been expected to have been given.

The VNPI was seen to affect the chances of getting planned (or actual) adjuvant RT in the univariate analysis, suggesting that clinicians may be starting to use this scoring system in routine practice to assist in decision making. It is possible that the VNPI not being statistically significantly in the multivariate model, after adjustment for size and grade, is an artefact of it being included in the model along with the factors from which it is derived. As such, it has a higher percentage of cases with unknown VNPI (16.8%) compared to its components (1.0% for grade, 4.6% for size and 12.3% for margin width). Additionally, the lack of an effect for margins of width <1 mm may have weakened the relationship. However, given the strong relationship observed in the univariate model between the VNPI and whether or not a patient was planned to receive RT, it seems reasonable to assume that when all of the components are known, and thus a VNPI can be derived, that this single prognostic indicator score would have been used to determine the decision about giving RT or not.

There was a very wide variation in the use of adjuvant RT by individual breast units, with some referring all patients treated by BCS for adjuvant RT and some referring none. Although it should be acknowledged that the available evidence concerning the utility of adjuvant RT following BCS for DCIS does allow some interpretational variation and does not permit a definitive policy to be formulated, such a wide variation in routine practice is of concern.

Data from the prospective randomised controlled trials (Fisher *et al*, 1998; Julien *et al*, 2000; UK Coordinating Committee on Cancer Research (UKCCCR) DCIS Working Party, 2003) concerning the use of adjuvant RT have been available for some years; for example, the UK DCIS I trial (UKCCCR DCIS Working Party, 2003) to which many of the breast units participating in the Sloane Project contributed, was published 3 years ago. These, albeit relatively few, trials have shown that adjuvant RT halves the risk of local recurrence.

It is possible that the variation in use of RT reflected differences in perception of the risk–benefit relationships of this treatment given that there is no evidence of any impact of adjuvant therapy for DCIS on breast cancer mortality but – given the pressures on RT facilities in the UK (Dodwell and Crellin, 2006) – some clinicians may feel that priority for patients should be given to those with invasive disease or at least those at highest risk of recurrence and therefore decisions concerning the use of RT for DCIS may reflect local RT capacity.

There is a clear need to establish a coherent approach to the management of DCIS. Evidence from clinical trials and high-quality epidemiological studies is required to support the approach of omitting adjuvant RT for low-risk groups and, in particular, to identify the characteristics of such groups robustly. Uniform standards of surgical excision and pathological measurement of distance to surgical margins are required. Similarly there are no agreed methodologies, scoring systems or cutoffs for the assessment of hormone receptor status in DCIS in the United Kingdom. Ongoing prospective trials, as well as the Sloane Project, will aid in the achievement of these goals and, it is to be hoped, will provide a better understanding of treatment effectiveness for this increasingly commonly detected disease, thus allowing a more consistent approach to patient management.

## Figures and Tables

**Table 1:** Variation with pathological factors in the planned and actual use of adjuvant RT for DCIS patients treated with breast-conserving surgery (BCS)

			**Radiotherapy treatment**											
			**RT planned**		**RT planned and given**		**RT planned but not given**		**RT planned but u/k if given**		**RT not planned**		**RT unknown if planned**	
**Pathological factor**	**Total no. of cases**	**% cases**	**No.**	**%**	**No.**	**%**	**No.**	**%**	**No.**	**%**	**No.**	**%**	**No.**	**%**
*Margins*														
>or=10 mm	417	36.6	230	55.2	206	49.4	17	4.1	7	1.7	180	43.2	7	1.7
5–9.99 mm	226	19.8	127	56.2	119	52.7	4	1.8	4	1.8	98	43.4	1	0.4
1–4.99 mm	240	21.1	155	64.6	137	57.1	10	4.2	8	3.3	83	34.6	2	0.8
<1 mm	117	10.3	68	58.1	63	53.8	3	2.6	2	1.7	49	41.9	0	0.0
Unknown margins	140	12.3	71	50.7	60	42.9	5	3.6	6	4.3	67	47.9	2	1.4
Total	1140	100.0	651	57.1	585	51.3	39	3.4	27	2.4	477	41.8	12	1.1
														
*Tumour size*														
<15 mm	678	59.5	307	45.3	269	39.7	24	3.5	14	2.1	362	53.4	9	1.3
16–40 mm	375	32.9	293	78.1	270	72.0	13	3.5	10	2.7	80	21.3	2	0.5
>40 mm	35	3.1	29	82.9	28	80.0	1	2.9	0	0.0	6	17.1	0	0.0
Unknown size	52	4.6	22	42.3	18	34.6	1	1.9	3	5.8	29	55.8	1	1.9
Total	1140	100.0	651	57.1	585	51.3	39	3.4	27	2.4	477	41.8	12	1.1
														
*Comedo necrosis*														
Not present	392	34.4	131	33.4	112	28.6	14	3.6	5	1.3	256	65.3	5	1.3
Present	720	63.2	503	69.9	460	63.9	23	3.2	20	2.8	210	29.2	7	1.0
Unknown necrosis	28	2.5	17	60.7	13	46.4	2	7.1	2	7.1	11	39.3	0	0.0
Total	1140	100.0	651	57.1	585	51.3	39	3.4	27	2.4	477	41.8	12	1.1
														
*Nuclear grade*														
Low	141	12.4	25	17.7	20	14.2	2	1.4	3	2.1	114	80.9	2	1.4
Intermediate	368	32.3	167	45.4	141	38.3	17	4.6	9	2.4	197	53.5	4	1.1
High	620	54.4	453	73.1	419	67.6	19	3.1	15	2.4	161	26.0	6	1.0
Unknown grade	11	1.0	6	54.5	5	45.5	1	9.1	0	0.0	5	45.5	0	0.0
Total	1140	100.0	651	57.1	585	51.3	39	3.4	27	2.4	477	41.8	12	1.1
														
*Van Nuys Score*														
3,4	236	20.7	64	27.1	52	22.0	9	3.8	3	1.3	168	71.2	4	1.7
5,6,7	673	59.0	459	68.2	423	62.9	20	3.0	16	2.4	209	31.1	5	0.7
8,9	39	3.4	31	79.5	30	76.9	1	2.6	0	0.0	8	20.5	0	0.0
Unknown Van Nuys	192	16.8	97	50.5	80	41.7	9	4.7	8	4.2	92	47.9	3	1.6
Total	1140	100.0	651	57.1	585	51.3	39	3.4	27	2.4	477	41.8	12	1.1

BCS=breast-conserving surgery; DCIS=ductal carcinoma *in situ*; RT=radiotherapy.

**Table 2:** Unadjusted and adjusted odds ratios of receiving planned RT for DCIS patients having breast-conserving surgery (BCS)

		**RT planned**							
				**Unadjusted**			**Adjusted**		
**Pathological factor**	**Total no. of cases**	**No.**	**%**	**ORs**	**CI**	***P*-value**	**ORs**	**CI**	***P*-value**
									
*Margins*						0.07			0.09
>or=10 mm	417	230	55.2	1.0	—		1.0	—	
5–9.99 mm	226	127	56.2	1.04	0.75, 1.45		1.03	0.70, 1.52	
1–4.99 mm	240	155	64.6	1.48	1.07, 2.06		1.66	1.12, 2.45	
<1 mm	117	68	58.1	1.13	0.75, 1.71		1.00	0.58, 1.72	
Unknown margins	140	71	50.7	0.84	0.57, 1.23		0.93	0.32, 2.76	
Total	1140	651	57.1						
									
*Tumour size*						<0.001			<0.001
<15 mm	678	307	45.3	1.0	—		1.0	—	
16–40 mm	375	293	78.1	4.32	3.24, 5.76		3.50	2.50, 4.90	
>40 mm	35	29	82.9	5.84	2.39, 14.25		5.71	2.16, 15.13	
Unknown size	52	22	42.3	0.89	0.50, 1.57		1.06	0.40, 2.86	
Total	1140	651	57.1						
									
*Comedo necrosis*						<0.001			<0.001
Not present	392	131	33.4	1.0	—		1.0	—	
Present	720	503	69.9	4.62	3.55, 6.01		2.05	1.45, 2.89	
Unknown necrosis	28	17	60.7	3.08	1.40, 6.76		2.28	0.90, 5.75	
Total	1140	651	57.1						
									
*Nuclear grade*						<0.001			<0.001
Low	141	25	17.7	1.0	—		1.0	—	
Intermediate	368	167	45.4	3.86	2.39, 6.22		2.55	1.52, 4.26	
High	620	453	73.1	12.59	7.89, 20.08		6.56	3.65, 11.80	
Unknown grade	11	6	54.5	5.57	1.57, 19.69		3.48	0.67, 18.13	
Total	1140	651	57.1						
									
*Van Nuys score*						<0.001			0.75
3,4	236	64	27.1	1.0	—		1.0	—	
5,6,7	673	459	68.2	5.76	4.15, 8.02		0.89	0.54, 1.49	
8,9	39	31	79.5	10.41	4.55, 23.85		0.52	0.16, 1.70	
Unknown Van Nuys	192	97	50.5	2.74	1.83, 4.11		0.88	0.27, 2.86	
Total	1140	651	57.1						

BCS=breast-conserving surgery; DCIS=ductal carcinoma *in situ*; RT=radiotherapy.
